# Efficacy of BrighterSide, a Self-Guided App for Suicidal Ideation: Randomized Controlled Trial

**DOI:** 10.2196/55528

**Published:** 2024-03-18

**Authors:** Natasha Josifovski, Michelle Torok, Philip Batterham, Quincy Wong, Joanne R Beames, Adam Theobald, Sarah Holland, Kit Huckvale, Jo Riley, Nicole Cockayne, Helen Christensen, Mark Larsen

**Affiliations:** 1 Black Dog Institute, Randwick, Australia; 2 The University of New South Wales, Sydney, New South Wales, Australia; 3 Centre for Mental Health Research, Research School of Population Health, Australian National University, Canberra, Australia; 4 School of Psychology, Western Sydney University, Sydney, Australia; 5 Centre for Digital Transformation of Health, University of Melbourne, Melbourne, Australia; 6 Coordinaire, Fairy Meadow, Australia

**Keywords:** suicidal ideation, suicide prevention, digital health, clinical trial

## Abstract

**Background:**

Self-guided digital interventions can reduce the severity of suicidal ideation, although there remain relatively few rigorously evaluated smartphone apps targeting suicidality.

**Objective:**

This trial evaluated whether the BrighterSide smartphone app intervention was superior to a waitlist control group at reducing the severity of suicidal ideation.

**Methods:**

A total of 550 adults aged 18 to 65 years with recent suicidal ideation were recruited from the Australian community. In this randomized controlled trial, participants were randomly assigned to receive either the BrighterSide app or to a waitlist control group that received treatment as usual. The app was self-guided, and participants could use the app at their own pace for the duration of the study period. Self-report measures were collected at baseline, 6 weeks, and 12 weeks. The primary outcome was severity and frequency of suicidal ideation, and secondary outcomes included psychological distress and functioning and recovery. Additional data were collected on app engagement and participant feedback.

**Results:**

Suicidal ideation reduced over time for all participants, but there was no significant interaction between group and time. Similar improvements were observed for self-harm, functioning and recovery, days out of role, and coping. Psychological distress was significantly lower in the intervention group at the 6-week follow-up, but this was not maintained at 12 weeks.

**Conclusions:**

The BrighterSide app did not lead to a significant improvement in suicidal ideation relative to a waitlist control group. Possible reasons for this null finding are discussed.

## Introduction

One in 6 adults experience serious thoughts of suicide during their lives, with an estimated prevalence of 3.4% of adults experiencing suicidal ideation in a 12-month period [1]. Data from the World Health Organization suggest that those with suicidal ideation are 10 times more likely to make a suicide attempt across their lifetime than those without suicidal ideation [2]. In recent years, there has been a shift in how people seek mental health support for a suicidal crisis. For example, during the 2021-2022 financial year, Lifeline—an Australian crisis service—answered 1,142,234 calls, a 56% increase since 2019 [3]. At the same time, hospitals in Australia experienced a 14.3% reduction in mental health–related presentations [4]. One potential way to offset the high demand for crisis support services is to make self-guided digital health interventions publicly available for those in distress. These interventions have the potential to increase access to mental health care and enhance the capacity of mental health systems to respond to persons in crisis by offering high-fidelity, evidence-based therapeutic support, anonymously and at low to no cost, which can be readily accessed anywhere [5,6].

Meta-analytic evidence suggests that digital health interventions can effectively reduce the severity of suicidal thoughts [7]. Those interventions that specifically target suicidality are more effective than generalized mental health apps for reducing suicide-related outcomes [7]. However, despite the huge potential for self-guided smartphone interventions to address service access gaps for those experiencing suicidal distress, there are currently few digital interventions targeting suicidal ideation that have been rigorously tested in randomized controlled trials (RCTs). Even fewer of these have been in general adult populations.

One exception to the above is the web-based self-help program Living with Deadly Thoughts, which was adapted from the Dutch program Living Under Control [8]. Drawing from principles of cognitive behavioral therapy and dialectical behavior therapy, this RCT involved access to 6 online modules for community-recruited adults experiencing suicidal ideation. While the Dutch program found a small but significant effect in reducing suicidal thoughts, the English-adapted Living with Deadly Thoughts program found no difference between intervention and control groups in an Australian study [8]. One possibility for this discrepancy may be that the English study was underpowered to detect an effect size that would be comparable with the Dutch version. However, there may be additional nuances given the difference in the recruited population—the Dutch study included only participants who experienced mild to moderate suicidal thoughts (defined as a score between 1 and 26 on the Beck Scale for Suicidal Ideation) and who were not severely depressed (defined as a scores greater than 39 on the Beck Depression Inventory). Conversely, Living with Deadly Thoughts did not include any cutoffs for either depression or suicidal thoughts as exclusion criteria. An effect on suicidal ideation could therefore be dependent on the severity of suicidal thoughts, in that those with thoughts that are more severe may not benefit from the modules involved. Alternatively, or as well as this, the program may involve other factors that are not generalizable, and an effective program to reduce suicidal ideation may require greater insight from those with lived experience.

In the context of these gaps, we developed BrighterSide. BrighterSide is a self-guided app based on a mix of cognitive behavioral therapy and dialectical behavior therapy, alongside elements of acceptance and commitment therapy and positive psychology. These therapeutic approaches have previously been demonstrated to be efficacious in reducing suicidal ideation when delivered through web-based programs [9,10]. The app involves 5 modules that each contain activities to encourage the user to develop and practice strategies to manage their suicidal thoughts. None of the activities last more than 5 minutes and users have complete control over which modules and activities they wish to complete. Furthermore, the app was co-designed with lived experience advisors in order to consider how best to maximize engagement and how best to support those with suicidal ideation or behaviors (see Torous et al [11] for a discussion on how co-design with consumers may enhance app engagement).

This study aimed to determine if those using the BrighterSide app would have a greater reduction in suicidal thoughts relative to the waitlist control group in a community trial in the adult population. We hypothesized that those in the intervention group would demonstrate significantly lower suicidal ideation, the primary outcome, at 6 and 12 weeks after baseline compared to a waitlist control group. We also hypothesized that the intervention group would report fewer incidents of self-harm and suicide attempts at 6 and 12 weeks after baseline and that the intervention group would report greater improvements in their ability to cope at 6 and 12 weeks after baseline.

## Methods

### Ethical Considerations

This study is reported as per the Consolidated Standards of Reporting Trials (CONSORT; Checklist 1) guideline (complete supplementary information is provided in Multimedia Appendix 1). The trial protocol was approved by the University of New South Wales Human Research Ethics committee (HC210196) and prospectively registered on the Australian New Zealand Clinical Trials Registry (ACTRN12621000712808).

### Trial Design

The trial was a single-blind, 2-arm parallel RCT. Participants were randomized 1:1 between intervention and waitlist-control groups. Researchers were blind to group allocation. Those allocated to the intervention group received immediate access to the BrighterSide app upon completing the baseline survey. Those allocated to the control group received access to the app at the end of the trial.

### Participants

Individuals were eligible for the study if they were (1) aged 18 to 65 years, (2) had experienced suicidal ideation within the previous 2 weeks, (3) owned an iPhone (with iOS 13.0 or higher) or Android (with Android 6.0 or higher) smartphone, (4) were fluent in English, and (5) currently lived in Australia. There were no specific exclusion criteria as this enabled a more heterogeneous sample.

Recruitment took place in 2 waves: the first was from June 30, 2021, to July 23, 2021, and the second was from September 20, 2021, to September 24, 2021 (see the Sample Size section). All data collection was completed by December 24, 2021. Participants were recruited via multiple channels. First, the trial was advertised on the Black Dog Institute (BDI) website. Second, recent visitors to the Black Dog Institute Online Clinic (a free mental health assessment tool) who had indicated recent suicidal ideation and consented to be contacted for future research opportunities were sent an invitation email by the clinic team. Third, the study was advertised on Facebook and Instagram via the BDI and Lifeline social media channels and paid advertising.

All individuals who responded to a study advertisement first completed an online screening questionnaire to determine eligibility. Those who screened as ineligible were redirected to a web page with information on crisis services. Eligible participants were presented with the participant information statement and digital consent form. Consenting participants then completed baseline questionnaires online via BDI’s bespoke trial software, upon completion of which participants were randomized. Randomization was performed by the trial management software using a block size of 4. Participants were notified of group allocation, but investigators remained blinded.

Participants in the intervention group were sent an email on baseline completion with a link to download the BrighterSide app from the Apple App Store or Google Play Store, along with a unique link that provided access to the app content. This prevented use of the app by users who were not registered to the trial. There was no specific timeframe to download the app, nor were any reminders sent if participants did not download it. Participants in the control group were sent an email with the link to access the app on completion of the final 12-week questionnaire (or, if not completed, at 13 weeks). Participants did not receive reimbursement or incentives to participate in the study. Participants were permitted to engage in other treatment, preexisting or new, while participating in the study.

### Intervention

BrighterSide is a self-guided smartphone app for adults experiencing suicidal ideation. The app was designed to help users develop and practice strategies to manage suicidal thoughts and was derived from content from the Living with Deadly Thoughts online program [8]. A multidisciplinary team of clinicians, researchers, lived experience advisors, designers, and developers engaged in a human-centered design process to update the original content into a more engaging form, ensuring the language, design, and user journey within the app were clear, simple, and supportive for adults experiencing suicidal thinking. This multidisciplinary collaboration was engaged across all functions of the app, including the safety planning and check-in features.

The app contained 5 modules: Understand your Thoughts, Prevent a Crisis, Navigate your Emotions, Navigate your Thoughts, and Plan for the Future (see Table S1 in Multimedia Appendix 1 [Supplementary-material SAP1][Supplementary-material SAP1][Supplementary-material SAP1]for brief details of each module, and Multimedia Appendix 2 for selected screenshots of each module). Each module contained interactive activities (eg, guided breathing). The content was based on cognitive behavioral therapy and dialectical behavior therapy, with elements of acceptance and commitment therapy and positive psychology. Users could access content in any order they wished or could choose a guided option that progressed through the modules in a specific order. The app included a safety planning function where users could list warning signs, helpful techniques, and supportive contact details and share them with others via email. The app also included a daily “check-in” feature that asked whether the user was safe and linked them to their safety plan and crisis contact numbers. Finally, in addition to the modules, the app also included “distraction activities,” such as Bubble Pop, a built-in game simulating the motion of popping bubble wrap, and “calming activities,” such as guided mindfulness recordings. Participants were free to engage with the app in whichever way they chose, including after the final data collection at 12 weeks, and the trial did not mandate frequency or patterns of use. A guided option was available if participants preferred, which allowed them to prioritise modules in a specific order depending on their main concern. The intervention did not include reminders to use the app. Participants in the waitlist control group received an email with details of crisis support services, and access to the app was granted after the 12-week study period.

### Outcomes

Standard demographic data were collected at baseline, with outcome measures collected at baseline, 6 weeks, and 12 weeks. Self-report questionnaires were administered online via the BDI’s Research Engine platform, and participants were sent a link via email at 6 and 12 weeks to complete the follow-up time points.

The primary outcome was the frequency and severity of suicidal ideation, as measured by the Suicidal Ideation Attributes Scale (SIDAS) [12]. This comprises 5 items measuring frequency of ideation, controllability, closeness to attempt, level of distress, and the impact on daily functioning. The total score is in the range 0 to 50, with higher scores indicating higher levels of suicidal ideation. Scores of 21 or higher are indicative of high risk for suicidal behavior [12]. The Cronbach α for this study was α=.803.

Secondary outcomes included self-harm behavior, coping strategies (abbreviated Coping Orientation to Problems Experienced Inventory; Brief-COPE [13]) functioning and recovery (Functioning and Recovery Scale; FRS [14]), psychological distress (Distress Questionnaire-5; DQ5 [15]), help-seeking (modified Actual Health-Seeking Questionnaire; ASHQ [16]), and days out of role (WHO Disability Assessment Schedule; WHODAS–1 item [17]). The distress, functioning and recovery, and days out of role measures were added following lived experience consultation—these measures were added following initial registration of the trial, but prior to recruitment opening.

At 6 weeks, participants in the intervention group completed additional measures in relation to the app: appropriateness of intervention (Implementation Appropriateness Measure; IAM [18]), the Digital Working Alliance Inventory (DWAI [19]) and a bespoke questionnaire seeking feedback on the BrighterSide app. Participants in the intervention group were also invited on completion of the 12-week measures to participate in a semistructured interview to provide detailed feedback on their experience using BrighterSide.

### Safety Monitoring

Data on adverse events and serious adverse events related to suicidal ideation and suicide attempts, respectively, were routinely collected at all time points though the self-report outcome measures already described. Specifically, severe suicidal ideation was indicated by a total score of 21 or higher on the SIDAS, and a recent suicide attempt was marked by either a self-report of 1 or more suicide attempts in the past 6 weeks (in the self-harm behavior questionnaire), or as indicated by a score of 10 (“I have made an attempt”) on question 3 of the SIDAS.

If a participant indicated they had recently attempted suicide (as described above), an email was automatically sent to them with support contact details and to arrange for a follow-up phone call by the research team. If a response was not received within 1 business day, a second email was sent to the participant. Follow-up phone calls ensured that the participant was safe and offered a referral to Lifeline, a telephone crisis support service who would be able to call the participant and offer specialized support. In the case of the intervention group, participants were also routinely asked during questionnaires if their suicidal ideation and/or suicide attempt was related to use of the BrighterSide app. If they indicated yes, the follow-up call would be identical to that described above, with an addition to seek clarity on if and how use of the app contributed to their suicidal thoughts or behaviours.

### Patient and Public Involvement

The BDI’s lived experience advisory team collaborated on the content and design of the app. Furthermore, the lived experience advisors were consulted on the trial design and recommended including outcome measures related to functioning and recovery (measured with the FRS), psychological distress (measured with the DQ5), and days out of role (measured with WHODAS-1).

### Sample Size

The initial recruitment target for the trial was 394 participants, with 197 participants per arm. This allowed for detection of a small to medium effect size (*d*=0.3) in the primary outcome (severity of suicidal ideation) between the intervention and control arms with 80% power (α=.05), allowing for 40% attrition at postintervention follow-up. This was informed by 3 previous trials that incorporated the underlying intervention content in a web-based program, reporting a pooled effect size of 0.31 and average attrition rate of 35% [8-10]. After observing a higher than estimated attrition rate at the 6-week follow-up (n=223; 56.6%), the recruitment target was raised to 546 (n=273 per arm) to maintain statistical power.

### Statistical Methods

Demographic and clinical characteristics were compared between study arms using 2-sided independent sample *t* tests, *χ*^2^ tests, or the Fisher exact test. Mixed model repeated measures (MMRM) analyses with maximum likelihood estimation and an unstructured covariance matrix were used to evaluate the efficacy of the BrighterSide app relative to the control condition. The primary outcome was severity of suicidal ideation as assessed by the SIDAS over time (baseline to 6 weeks; baseline to 12 weeks). The mixed model approach incorporates all available data, including participants with missing follow-up data points, under the missing-at-random assumption that is robust to data that are missing contingent on observed variables. Analyses were performed under the intention-to-treat principle by a statistician who was blinded to group allocation. Analyses of secondary outcomes used the same methods for continuous outcomes. The frequency of adverse events, including severe suicidal ideation, recent self-harm, and recent suicide attempts, were compared between groups using a *χ*^2^ test of independence.

Descriptive statistics were used to evaluate use (indicated by the number of modules completed, recorded using app analytics) of the BrighterSide app by participants in the intervention condition. Interview data collected from a subset of participants in the intervention group were analyzed thematically by one of the authors [20]. An inductive approach, independent of a theoretical confirmative method, was used to identify and group themes. Themes were refined to determine the final coding framework.

### Post Hoc Analysis

A post hoc analysis using logistic regression was performed to assess whether follow-up attrition rates at 6 and 12 weeks after baseline could be predicted by any factors measured at baseline.

## Results

### Recruitment and Baseline Characteristics

A total of 795 participants were assessed for eligibility, of whom 550 were randomized (see Figure S1 in Multimedia Appendix 1). A total of 275 participants were randomized to each group. All participants were analyzed under the intention-to-treat principle, except 1 (in the intervention group) who withdrew and requested that their data not be retained. Baseline characteristics for participants are presented in Table 1; the groups did not differ across any measure.

**Table 1.** Baseline characteristics and clinical outcomes for each group. Significance values refer to comparisons of the 2 groups using a 2-sided independent-sample *t* test, except where footnoted.

		Total (n=549)	BrighterSide (n=274)	Control (n=275)	*P* value
**Characteristics**					
	Female, n (%)	399 (72.7)	203 (74.1)	196 (71.3)	.84^a^
	Age (years), mean (SD)	39.1 (13.5)	39.1 (13.5)	39.1 (13.5)	.98
**Actual Help-Seeking Questionnaire**					
	Mental health help sought in past 3 months, n (%)	453 (82.5)	226 (82.5)	227 (82.6)	.54^a^
**Suicidal Ideation Attributes Scale**					
	Mean score (SD)	25.8 (10.1)	25.7 (10.3)	25.8 (10)	.89
	Score ≥21, n (%)	366 (66.7)	178 (64.9)	188 (68.4)	.26^a^
**Self-harm (%)**					
	Have you ever harmed yourself on purpose? (score >0)	416 (75.8)	208 (75.9)	208 (75.6)	.51^a^
	In the last six weeks have you harmed yourself on purpose? (score >0)	202 (36.8)	101 (36.9)	101 (36.7)	.54^a^
	Have you ever attempted to take your own life? (score >0)	291 (53)	152 (55.5)	139 (50.6)	.14^a^
	In the last six weeks have you attempted to take your own life? (score >0)	38 (6.9)	19 (6.9)	19 (6.9)	.45^a^
Functioning and Recovery Scale score, mean (SD)		13.3 (2.4)	13.3 (2.4)	13.4 (2.3)	.55
Distress Questionnaire-5 score, mean (SD)		19.2 (2.8)	19.3 (2.7)	19.2 (2.9)	.67
WHO Disability Assessment Schedule (1 item for days out of role) score, mean (SD)		10.1 (8.9)	10.4 (9.2)	9.9 (8.7)	.45
**Abbreviated Coping Orientation to Problems Experienced Inventory score, mean (SD)**					
	Problem-focused score	18.4 (4.9)	18.3 (5.1)	18.4 (4.8)	.79
	Emotion-focused score	27.6 (4.9)	27.7 (5.2)	27.5 (4.7)	.53
	Avoidant-focused score	18.2 (3.6)	18.3 (3.6)	18.1 (3.7)	.69

a *P* value refers to the *Χ*2 or Fisher exact test.

### Primary and Secondary Outcomes

**Figure 1.**
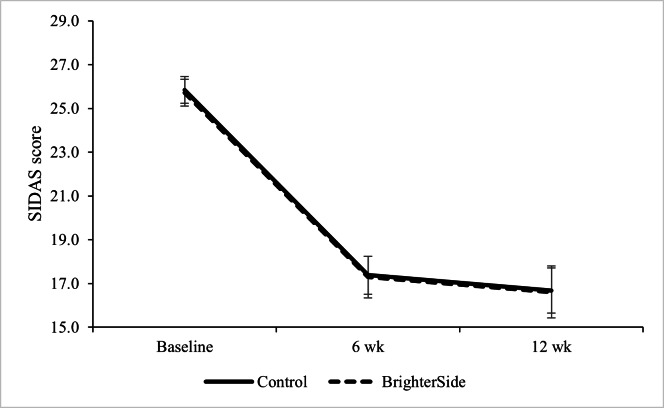
Suicidal ideation (Suicidal Ideation Attributes Scale; SIDAS) scores by group at baseline, 6 weeks, and 12 weeks. Error bars represent 1 SE.

The mixed effects models for the primary and secondary outcomes are shown in Table 2. The main effect of time was significant for suicidal ideation, functioning and recovery, days out of role, psychological distress, problem-focused coping, and avoidant coping. There were no main effects for condition for any of the measures, and the time by group interaction was only significant for psychological distress. Residuals for SIDAS scores were nonnormal; however, results were identical under a negative binomial MMRM. Therefore, while the primary outcome, suicidal ideation, demonstrated a significant reduction over time, this did not significantly differ between groups (Figure 1). The Cohen *d* effect size for suicidal ideation between the intervention and control groups from baseline to 6 weeks was *d*=–0.03, and *d*=–0.15 from baseline to 12 weeks. Negative effect sizes favor the control group. See Table S2 in Multimedia Appendix 1 for summary of Cohen *d* effect sizes.

**Table 2.** Omnibus mixed model repeated measures ANOVA time (baseline; 6 weeks; 12 weeks) × group (BrighterSide; control).

		*F* test (*df*)	*P* value
**Suicidal Ideation Attributes Scale**			
	Time	124.11 (2,223.1)	<.001^a^
	Group	0.01 (1,404.3)	.92
	Time × group	0.00 (2,223.1)	.99
**Functioning and Recovery Scale**			
	Time	25.89 (2,242.7)	<.001^a^
	Group	0.02 (1,384.1)	.88
	Time × group	1.18 (2,247.7)	.31
**WHO Disability Assessment Schedule (1 item for days out of role)**			
	Time	13.69 (2,198.6)	<.001^a^
	Group	0.04 (1,375.9)	.83
	Time × group	1.05 (2,198.6)	.35
**Distress Questionnaire-5**			
	Time	47.04 (2,234.0)	<.001^a^
	Group	1.74 (1,396.3)	.18
	Time × group	3.62 (2,234.0)	.03^a^
**Self-harm**			
	Time	37.29 (2,214.8)	<.001^a^
	Group	0.00 (1,411.6)	.97
	Time × group	0.35 (2,214.8)	.70
**Brief-COPE**^b^ **(problem)**			
	Time	3.38 (2,221.4)	.01^a^
	Group	0.71 (1,380.3)	.40
	Time × group	1.03 (2,221.4)	.35
**Brief-COPE (emotional)**			
	Time	0.04 (2,225.7)	.96
	Group	2.11 (1,370.2)	.15
	Time × group	1.01 (2,225.7)	.36
**Brief-COPE (avoidant)**			
	Time	18.35 (2,225.6)	<.001^a^
	Group	1.24 (1,381.4)	.26
	Time × group	2.45 (2,225.6)	.08

a Significant at α=.05.

b Brief-COPE: abbreviated Coping Orientation to Problems Experienced Inventory.

**Figure 2.**
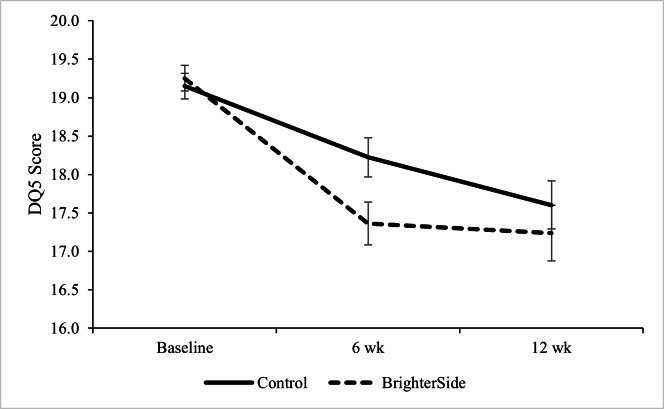
Distress (Distress Questionnaire-5; DQ5) scores by group at baseline, 6 weeks, and 12 weeks. Error bars represent 1 SE.

Follow-up analysis of the interaction between time and group in the DQ5 demonstrated a significant difference between the intervention and control groups at 6 weeks *(t*_244.60_=2.68, 95% CI 0.25-1.67; *P*=.01), where the BrighterSide group reported lower scores (mean 17.36, SE 0.27) than the control group (mean 18.22, SE 0.25; Cohen *d*=0.26). However, this difference was no longer significant at 12 weeks *(t*_189.61_=1.05, 95% CI –0.41 to 1.35; *P*=.29; Cohen *d*=−0.01) (Figure 2).

### Adverse Events

Comparison of the frequency of severe suicidal ideation, self-harm, and suicide attempts between the control and intervention groups yielded no significant differences at either 6 weeks or 12 weeks (Table S3 in Multimedia Appendix 1). Given the target population were people experiencing suicidal ideation at baseline, the frequency of adverse events is not remarkable at either follow-up time point.

### Attrition

We used a post hoc logistic regression analysis modeling predictors of attrition at 6 weeks and 12 weeks. SIDAS and FRS were chosen as variables to investigate for any potential moderating effects, rather than all surveys, as doing so may have violated the assumption of no multicollinearity (full results available in Table S4 in Multimedia Appendix 1). The only significant predictors of attrition were group at 12 weeks and the SIDAS × group interaction at 12 weeks. The interaction reflects a greater proportion of participants with lower levels of suicidal ideation (SIDAS scores of 0-20) dropping out in the intervention group (77/96, 80%) compared to the control group (48/87, 59%).

### User Engagement Outcomes

Analytic information regarding the frequency of use of the app was obtained. Overall, 188 intervention participants (n=275, 68.4% of the baseline intervention group) enrolled into the BrighterSide app, which is the initial onboarding task after downloading and opening the app. Table 3 presents descriptive statistics on module engagement across the course of the trial.

**Table 3.** Engagement with activities by module in the BrighterSide app (n=275).

	Module									
	Understand Your Thoughts		Prevent a Crisis		Navigate Your Emotions		Navigate Your Thoughts		Plan for the Future	
	No.^a^	%^b^	No.	%	No.	%	No.	%	No.	%
Engaged with ≥1 activity in module	100	36.4	58	21.1	57	20.7	40	14.5	47	17.1
Engaged with ≥50%of activities in module	95	34.5	57	20.7	33	12	13	4.7	27	9.8
Completed all activities in module	19	6.9	22	8	8	2.9	6	2.2	4	1.5

a No.: number of users.

b %: percentage of baseline sample randomised to the intervention group.

In addition to module engagement, we also reviewed engagement with distraction and calming activities. We found that 69 users (25.1% of the intervention group) engaged with at least 1 calming activity and 43 users (15.6% of the intervention group) engaged with at least 1 distraction activity. Given the low percentage of engagement with these activities, they were not considered further.

### Use and Acceptability Outcomes

Tables S5 and S6 in Multimedia Appendix 1 present the results of the appropriateness (IAM) and therapeutic alliance (DWAI) measures collected at the 6-week time point. The average of the appropriateness scales was 3.63 (SD 0.89) on a range of 1 to 5, indicating that the participants’ response to the app was positively skewed. This result is consistent with another mental health–oriented app using this same measure, which returned an average of 3.6 [21]; these authors regarded this as an indication of appropriateness. Each question on the DWAI averaged a response between 2.11 and 3.28 across all participants, on a scale of 1 (not at all) to 4 (completely), suggesting that typically, participants indicated they somewhat to mostly agreed with each statement.

Participants were also given a study-specific acceptability survey, where only 44 of 86 responders (51%) agreed the app met their needs, but 73 of those responders (85%) agreed it was easy to use (agreement was determined by a score of 5 or higher on a 7-point scale; see Table S7 in Multimedia Appendix 1 for full results). This survey also included questions regarding the use of the safety plan feature. This showed that 31 of the 86 (36%) responders to this question had filled out the safety plan, and 100% of these did so alone; 81% (25/31) did not share the safety plan with someone else, and the remaining 19% (6/31) did so with one other person. Finally, 58% (18) of those who completed their safety plan looked back on it. For those who did not fill out the safety plan, the most frequent responses when asked why were that they did not feel they had the time; they found it too confronting or overwhelming; they had completed a safety plan elsewhere; or they simply did not know how to navigate to it in the app.

### Semistructured Interviews

Of the 69 intervention participants who completed the 12-week follow-up, 6 agreed to be interviewed about their experiences of using the BrighterSide app. Themes and subthemes that emerged throughout the interviews are described in Table S8 in Multimedia Appendix 1. The most notable patterns were that participants found the distraction and mindfulness activities particularly useful, and that the check-in function alleviated the burdensomeness often associated with reaching out to support networks. At the same time, some participants said that while not all of the information in the modules was helpful to them, it may be useful for people who have little or no experience with professional mental health care. Participants felt that the safety plan was useful as a reference tool for when they may be entering a crisis, and that the ability to share this plan with others was helpful since they often are unable to recognize their own warning signs.

## Discussion

### Principal Findings

The primary objective of this study was to determine if use of the BrigherSide app significantly decreased suicidal ideation at 6 weeks and 12 weeks after baseline compared to a waitlist control group. While there was a significant overall decrease in suicidal ideation from baseline to 12 weeks, there was no difference between the 2 groups. However, the intervention group did report significantly lower psychological distress at 6 weeks compared to the control group, although this difference was no longer significant at 12 weeks. There was no difference in rates of adverse events (severe suicidal ideation, recent self-harm, or recent suicide attempts), nor were there any significant moderators of demographic variables (age and gender) on attrition.

There may be several possible reasons for the null effect on suicidal ideation. First, there was little engagement with the app. While 68% downloaded and completed onboarding of the app, only 36% of the intervention group engaged with at least one activity in the first module (Understand Your Thoughts; see Table 3). This reflects that participants in the intervention group were not exposed to the full anticipated benefit of BrighterSide. Indeed, only 51% of participants found the app had met their needs. This may be an artifact of the nonprescriptive approach to the intervention. Participants were able to access content in any order they wished, or, if they preferred, use a guided option. If participants were initially exposed to the first module and did not find it helpful, they may have been less inclined to engage with the remaining modules, which may have been more relevant to them. In a similar vein, it could be argued that, given the identified relationship between thwarted belongingness and suicidal ideation [22,23], placing the onus on the participant to choose their method of interaction without the possibility of social connection (ie, no contact with another person) may have been counterintuitive to the aim of reducing suicidal ideation. This may reflect the finding that only half of participants felt the app met their needs, and an investigation into the usefulness of an app with or without a social connection aspect, and the impact of this on app engagement, should be investigated in future research. In any case, the lack of engagement with the app would anticipate a null effect.

Second, a high proportion of participants (453 of 550 participants, over 80%) had recently sought professional help for their mental health, and the strategies provided in the app may therefore have been already known to participants. Third, the trial recruitment period occurred during a period of public health protections in the COVID-19 pandemic. While rates of suicidal ideation were high but stable during the period of this study [24], broader public mental health support during this period may have had a confounding effect on the trial. Finally, despite the extensive co-design process to engage people with lived experience and clinicians, the final intervention may not have achieved an optimal balance between therapeutic content and user engagement. Given the null effect of the BrighterSide app on suicidal ideation, the remainder of this discussion will evaluate the differences between BrighterSide and other digital interventions that have also aimed to reduce suicidal ideation.

### Psychological Distress

Participants in the BrighterSide group reported significantly lower distress at 6 weeks than did those in the control group. While this difference was not maintained at 12 weeks, it might indicate that use of the app provided useful tools to navigate psychological distress in the short term. This is noteworthy given that high psychological distress is evidenced to be related to high reports of suicidal ideation and suicide attempts [25]. Ameliorating psychological distress may therefore act as a protective factor, although this is not directly captured by the data. Given that this study indicates the two are not comorbid, further investigation into the relationship between psychological distress and suicidal ideation, with greater power, is warranted.

### Comparison of Therapeutic Models

The previous finding that the digital intervention Living with Deadly Thoughts did not significantly reduce suicidal ideation between groups should be discussed in the context of this study [8]. While BrighterSide is modeled on this previous intervention, one of the key differences was the co-design process involved in developing the content for BrighterSide. Incorporating consumers in the design process of these interventions was one method proposed by Torous et al [11] to enhance engagement. Despite this, BrighterSide did not see substantial app engagement, and was rated by almost half of participants to have not met their needs. However, it is known that suicide prevention apps are useful in reducing suicide ideation—for example, the LifeBuoy app saw a significant difference in suicide ideation for the intervention group compared to an active control group [26]. We therefore consider the differences in the therapeutic content involved in these 2 models.

BrighterSide contains modules based on a mix of cognitive behavioral therapy, dialectical behavior therapy, acceptance and commitment therapy, and positive psychology. The amalgamation of different therapeutic elements for a brief intervention such as BrighterSide may have lacked enough adherence to a particular model to see any benefit. Instead, a greater effect on suicidal ideation might be achieved with modules that adhere to one therapeutic model, such as dialectical behavior therapy, since it has been shown to have a great effect on reducing suicidal behavior (see Ougrin et al [27] for a review). The LifeBuoy study implemented an intervention that followed a dialectical behavior therapy model and demonstrated a significant reduction in suicidal ideation when compared to a control group. They also allowed flexibility with module use, but their implementation was prescriptive, so that one module had to be completed in order to unlock the next. Additionally, if there were greater coherence within the modules, perhaps participants would be more inclined to engage meaningfully. Given these results, and the null findings for BrighterSide, it may be beneficial in future research to adhere to a single therapeutic model, such as dialectical behavior therapy, to both enhance delivery of skills and to enhance engagement among participants.

### Limitations

The most considerable limitation in this study is the low engagement with the app itself, which inhibits the capacity to adequately assess the primary and secondary outcomes. While maintaining engagement with app use is a recognized issue in digital mental health [11], this study did take into consideration some factors to enhance usability via the co-design method. Additionally, the way the app conveyed information may not have been conducive to participants actually implementing the learned knowledge and skills from the modules, particularly given the brief nature of the app and the amalgamation of components from different therapeutic models. Future research should consider assessing the learnability of skills portrayed in the modules, to determine whether apps are able to implement behavioral change.

While the interviews with participants were mostly positive, interviews were conducted with participants who self-selected to participate in an interview after completing the 12-week follow-up. Therefore, the small number who self-selected were more likely to have actively engaged in the app, generally had greater motivation for improving mental health research, and may have already felt more positively about the app. Regardless, the interview outcomes were consistent with the results from the surveys on acceptability, functionality, and perceptions of BrighterSide.

### Conclusion

This study aimed to investigate the ability of an app, BrighterSide, to reduce suicide ideation. While there were no between-condition effects for suicidal ideation, the severity of psychological distress was significantly reduced in the intervention condition after having access to the app for 6 weeks, relative to the control group. Further work may be required to optimally incorporate effective therapeutic content with engaging user design.

## Supplementary material

10.2196/55528 Multimedia Appendix 1 Complete set of appendices.

10.2196/55528 Multimedia Appendix 2 BrighterSide mobile app screenshots.

10.2196/55528 Checklist 1 CONSORT-EHEALTH (Consolidated Standards of Reporting Trials of Electronic and Mobile Health Application and online Tele Health) checklist.

## Abbreviations

ASHQ: Actual Health-Seeking Questionnaire
BDI: Black Dog Institute
Brief-COPE: Coping Orientation to Problems Experienced Inventory
CONSORT: Consolidated Standards of Reporting Trials
DQ5: Distress Questionnaire-5
DWAI: Digital Working Alliance Inventory
FRS: Functioning and Recovery Scale
IAM: Implementation Appropriateness Measure
MMRM: mixed model repeated measures
RCT: randomized controlled trial
SIDAS: Suicidal Ideation Attributes Scale
WHODAS: World Health Organization Disability Assessment Schedule
