# Supplementary material for: Efficacy of BrighterSide, a Self-Guided App for Suicidal Ideation: Randomized Controlled Trial
Source: JMIR Ment Health. 2024 Mar 18;11:e55528. doi: 10.2196/55528 (PMC11004607; doi:10.2196/55528)
Supplement: Multimedia Appendix 1 [file mental-v11-e55528-s001.docx]

### Figure S1: Participant flow chart


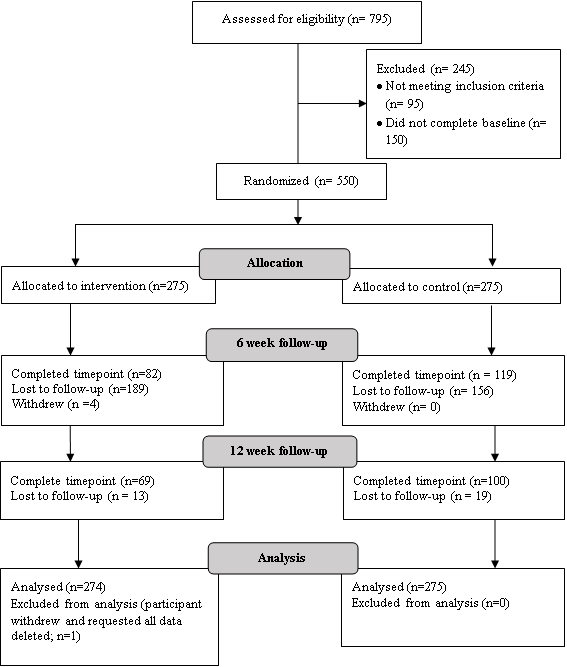


### Table S1: Title and brief description for each module in the BrighterSide app

| Title | Description |
| --- | --- |
| Module 1 – Understand your thoughts | Discusses why people have suicidal thoughts, how to identify common suicidal thoughts, and how using techniques like ‘worry time’, breathing exercises and mindfulness can help identify and then begin to manage these thoughts. |
| Module 2 – Prevent a crisis | Defines a suicidal crisis, teaches users to identify whether they are experiencing a crisis, defines the role and importance of a safety plan, and encourages users to create a safety plan using the app. |
| Module 3 – Navigate your emotions | Discusses how emotions can impact people and how individuals use different coping strategies, discusses feelings of guilt and worthlessness, discusses the importance of finding positivity and remembering good times. |
| Module 4 – Navigate your thoughts | Teaches users to identify unhelpful thinking habits, outlines common unhelpful thoughts, defines negative automatic thoughts, teaches users to challenge thinking habits. |
| Module 5 – Plan for the future | Discusses how to build resilience, the importance of focusing on your values and strengths, and goal setting. |

### Table S2. Cohen’s *d* effect sizes for observed differences between intervention and control groups, between baseline and 6 weeks, and baseline and 12 weeks.

|  | **Baseline – 6 Weeks** |  | **Baseline – 12 weeks** |
| --- | --- | --- | --- |
| SIDAS | -0.03 |  | -0.15 |
| FRS | 0.01 |  | -0.21 |
| WHODAS | 0.05 |  | -0.01 |
| DQ5 | 0.26 |  | -0.01 |
| Self-harm | 0.06 |  | -0.04 |
| Brief-COPE (Problem) | -0.13 |  | -0.19 |
| Brief-COPE (Emotional) | -0.06 |  | -0.19 |
| Brief-COPE (Avoidant) | -0.03 |  | -0.26 |

*Note.* Effect sizes were calculated using mean change in intervention group scores minus mean change in control group scores, divided by the pooled SD at baseline.

### Table S3. Frequencies and p-values for Chi square tests of independence between control and intervention groups for adverse events at 6 weeks and 12 weeks.

|  | **Timepoint** | | | | | | |
| --- | --- | --- | --- | --- | --- | --- | --- |
|  | **6 weeks** | | |  | **12 weeks** | | |
| **Adverse event type** | **Intervention** | **Control** | **p** |  | **Intervention** | **Control** | **p** |
| Severe suicidal ideation | 38 | 44 | 0.677 |  | 31 | 36 | 0.272 |
| Recent self-harm* | 36 | 39 | 0.478 |  | 27 | 31 | 0.328 |
| Recent suicide attempt* | 6 | 7 | 1.000 |  | 1 | 5 | 0.403 |

*Notes*. * = in the past 6 weeks.

### Table S4: Logistic regression odds ratios (OR) and significance values for each predictor variable on attrition at 6 weeks and 12 weeks. * denotes significant at α = .05.

|  | **Attrition at 6 weeks** | | **Attrition at 12 weeks** | |
| --- | --- | --- | --- | --- |
|  | **OR** | **P** | **OR** | **p** |
| **Age** | 1.001 | .939 | .991 | .183 |
| **Gender - Male** | 1.197 | .402 | 1.023 | .922 |
| **Gender - Female** | 1.302 | .614 | 1.226 | .706 |
| **Gender - Non-binary^1^** | .997 | .973 | .402 | .272 |
| **SIDAS** | 1.011 | .409 | 1.028 | .059 |
| **Group** | 1.922 | .174 | 5.440 | .001* |
| **FRS** | .946 | .192 | .964 | .416 |
| **SIDAS * Group** | .991 | .591 | .956 | .017* |

^1^Due to a low number of respondents in each, non-binary respondents were combined with those who indicated they used a different term to define their gender, or those who preferred not to answer.

### Table S5: Implementation Appropriateness Measure (IAM) Results

Questions are rated on a scale of 1 (completely disagree) to 5 (completely agree). The means and standard deviation for each item are reported.

| **Question** | **M** | **SD** |
| --- | --- | --- |
| BrighterSide seems fitting | 3.59 | 0.82 |
| BrighterSide seems suitable | 3.71 | 0.92 |
| BrighterSide seems applicable | 3.73 | 0.84 |
| BrighterSide seems like a good match | 3.48 | 0.99 |

### Table S6: Digital Working Alliance Inventory (D-WAI) Results

Questions are rated on a scale of 1 (not at all) to 4 (completely). The means and standard deviation for each item are reported.

| **Question** | **M** | **SD** |
| --- | --- | --- |
| I trust the app to guide me towards my personal goals | 2.21 | 0.78 |
| I believe the app tasks will help me address my problem | 2.11 | 0.72 |
| The app encourages me to accomplish tasks and make progress | 2.29 | 0.89 |
| I agree that the tasks within the app are important for my goals | 2.35 | 0.85 |
| The app is easy to use and operate | 3.28 | 0.77 |
| The app supports me to overcome challenges | 2.24 | 0.81 |

### Table S7: BrighterSide Feedback

Questions are rated on a scale of 1 (strongly disagree) to 7 (strongly agree). The means and standard deviation for each item are reported.

| **Question** | **M** | **SD** |
| --- | --- | --- |
| BrighterSide met my needs | 4.43 | 1.44 |
| BrighterSide was easy to use | 5.86 | 1.42 |
| The BrighterSide content was easy to read and understand | 6.02 | 1.03 |
| BrighterSide had the right amount of information | 5.05 | 1.49 |
| The BrighterSide content is relevant and useful | 5.31 | 1.42 |
| BrighterSide uses appropriate language and words | 5.98 | 1.13 |
| I was concerned about the privacy and safety of my data on this app | 2.25 | 1.76 |
| The app is a trustworthy source of mental health information | 5.73 | 1.24 |
| It was clear to me that the app would work | 4.53 | 1.54 |
| I don’t think an app can help me get better | 4.12 | 1.65 |
| I am already in treatment and don’t see the need for an app | 2.70 | 1.67 |
| It is embarrassing to have an app like this on my phone | 1.64 | 1.23 |
| I tried an app like this before and it did not help | 3.39 | 1.94 |
| There was enough guidance in the app to know what to do | 4.83 | 1.58 |
| There was enough feedback in the app | 4.35 | 1.51 |

### Table S8: Categories and themes that emerged during the thematic analysis of exit interviews. N = 6.

| **Theme and sub-themes** | **Example quotes** |
| --- | --- |
| **Positive responses to the app** | |
| Alleviates burdensomeness and ‘offloads’ feelings | You don't want to bother anyone. You don't think that anybody or listen. I suppose it really helped me that moment to just go through and read through the information and some of the activities... |
| Distraction activities allowed for something to focus on | You got ideas on what you can do and whatnot. Which is really quite nice 'cause when you're really stuck and you can't focus on anything, it kind of helps you try and direct thinking onto something now... |
| Comforting and validating | Even though there's nobody there, I felt that sort of made me feel better. I felt like I was sort of offloading how I was feeling or something, even though it wasn't really a person. |
| Motivating | …like about writing out things or journaling, or doing a little activity like the suggestions of activities outside of the app. I really liked that, so I suppose it's kind of motivated me...into wanting to do a bit more generally again, 'cause I used to do that and I forgot that I even had one. |
| Helped to understand own feelings | And I found it useful just like reading information to that, I understand how I was feeling and just distract from like being too in the feeling and put it trying to understand it better I suppose. |
| **Utility of the app** | |
| Some parts unhelpful or other support more helpful | You know it's one thing to know things, and another thing to be able to do them. |
| Information not new and/or might be more useful for people who are not already familiar with it | If it wasn't helpful for me, it would be helpful with somebody I thought. |
| Used app regularly in first few weeks | Almost every day for a time there. |
| App used as-needed | Actually I just really like have been able to have it on my phone and just having it there. So when I like scrolling past everything, I just say I better just check in or whatnot. |
| Did not complete modules or did not return to modules | The part I flicked through had some good resources on there. |
| Will continue to use app, or overall positive view of app functionality | I'll definitely keep using it if I'm able. |
| Safety plan helpful and/or shared safety plan with others | …like sometimes I'm not having a crisis like I'm not that bad but then you look at your warning signs [on the safety plan] and then you actually see it and you're like yeah, I probably am having a crisis right now |
| Did not complete safety plan or did not return to safety plan | I don't really worry about my safety. |
| Supported app being integrated into routine care as a supplement to other care | If it was integrated into other care I think it could be helpful for a lot of people. |
| Recommended changes to layout or functionality of app | ...it would be really good if you could turn on dark mode so it doesn't have the bright light 'cause I'm really sensitive to bright light... |
