# Supplementary material for: Efficacy of BrighterSide, a Self-Guided App for Suicidal Ideation: Randomized Controlled Trial
Source: JMIR Ment Health. 2024 Mar 18;11:e55528. doi: 10.2196/55528 (PMC11004607; doi:10.2196/55528)

**Supplemental File 2**

Selected screenshots of each module from the BrighterSide app.

**Module 1 - ‘Understand your thoughts’:**


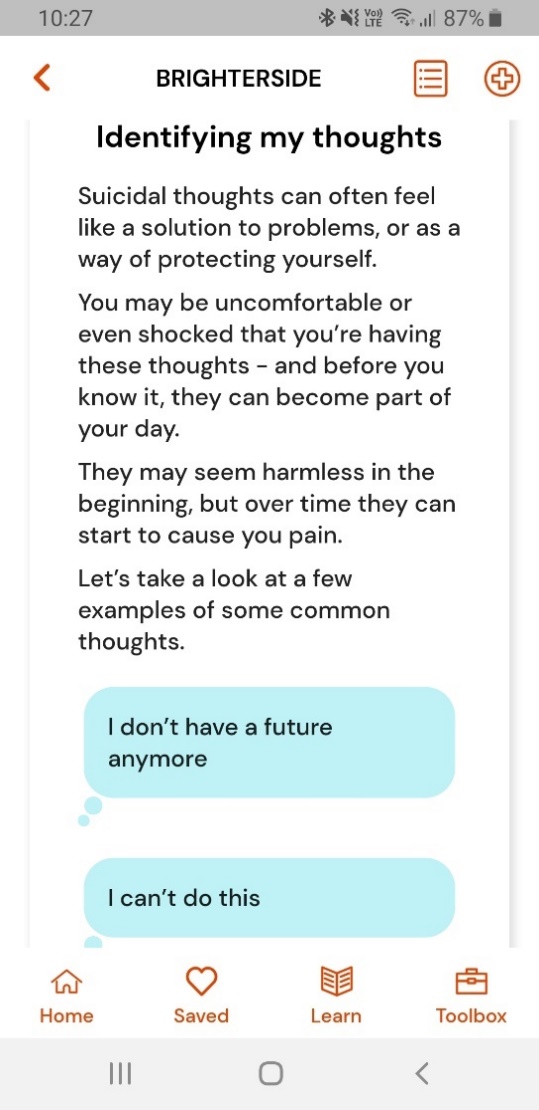

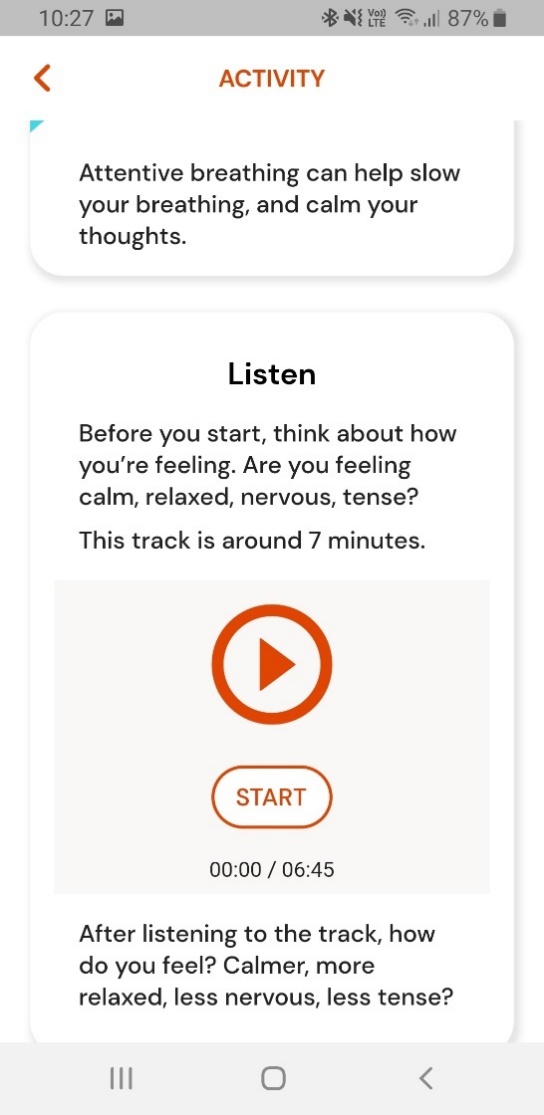


**Module 2 – ‘Prevent a crisis’:**


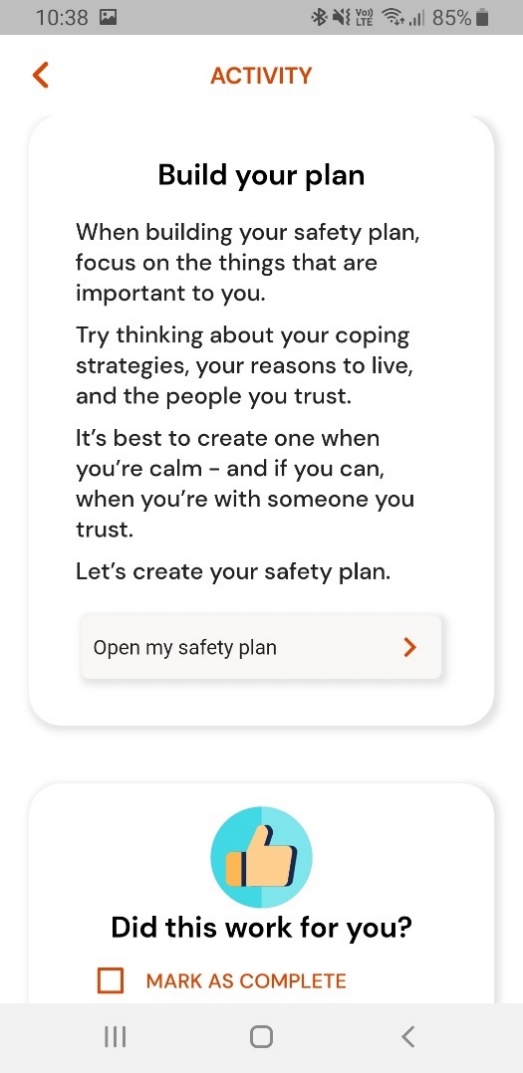

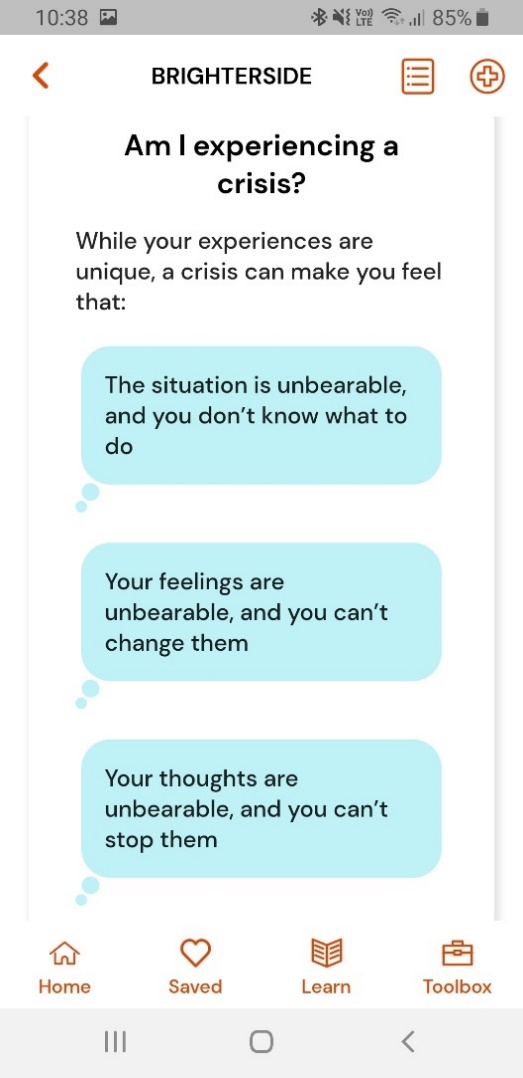


**Module 3 – ‘Navigate your emotions’:**


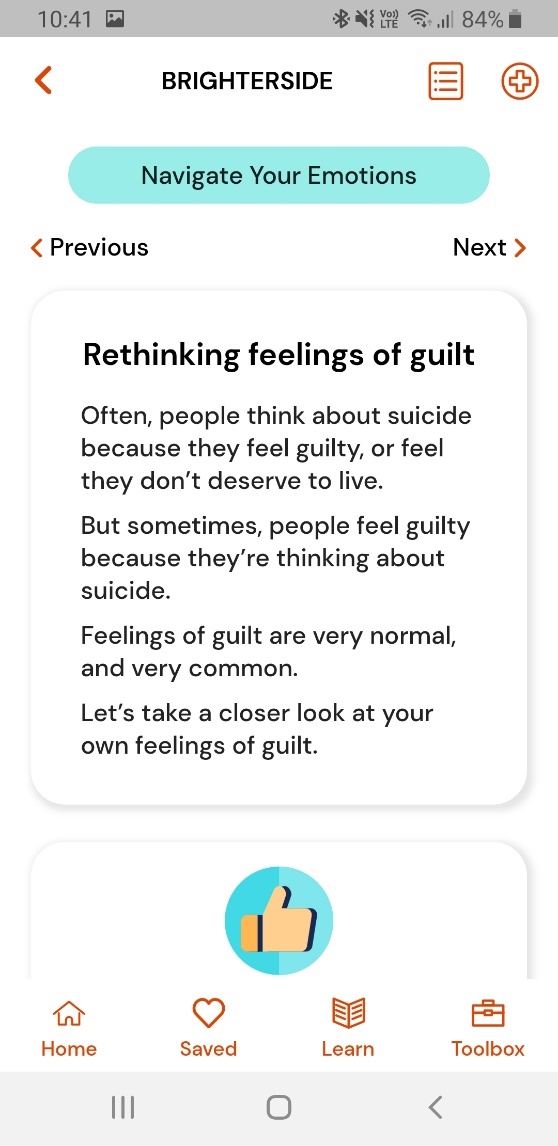

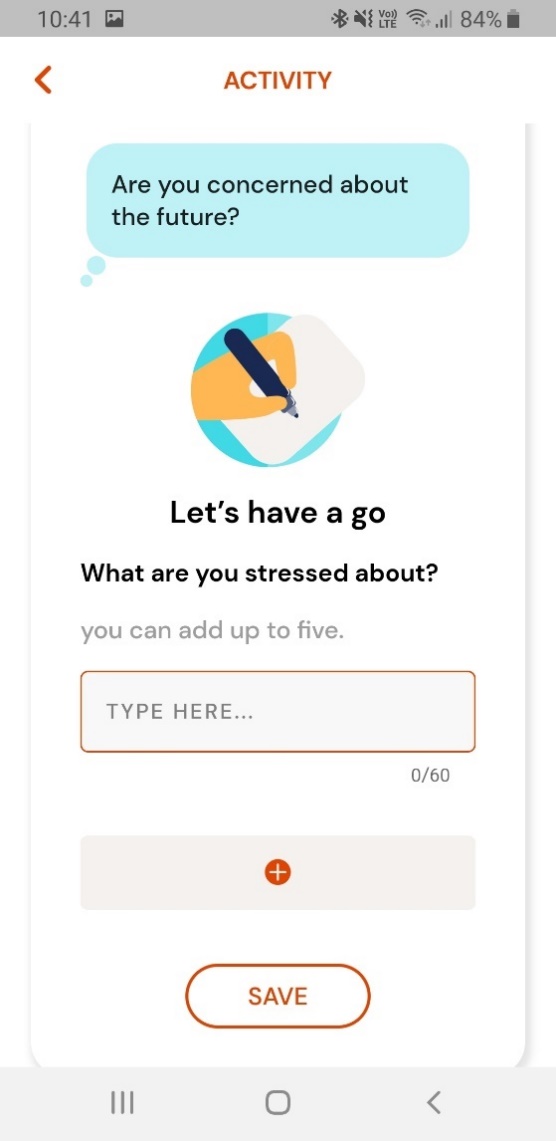


**Module 4 – ‘Navigate your thoughts’:**


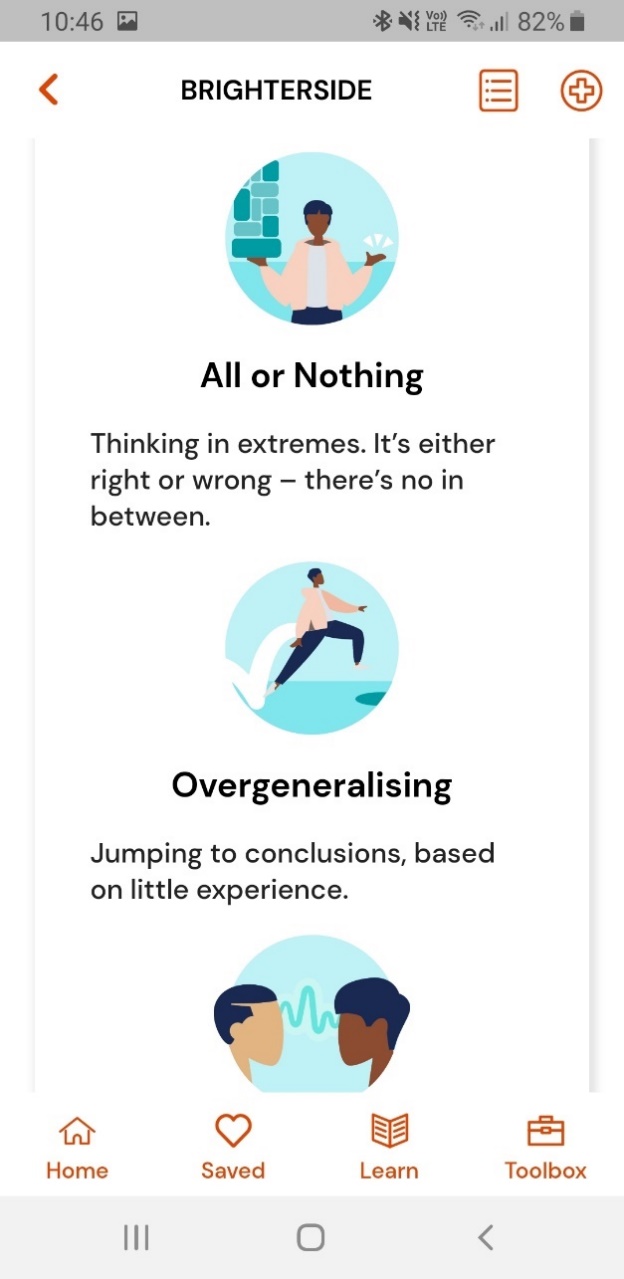

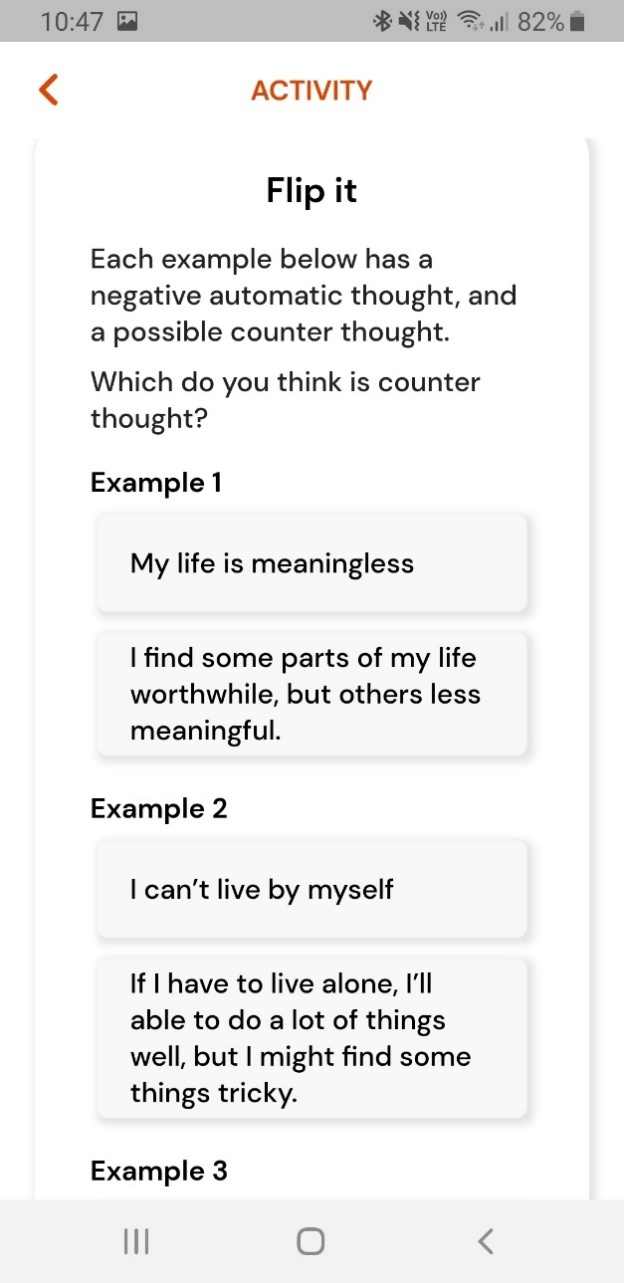


**Module 5 – ‘Plan for the future’:**


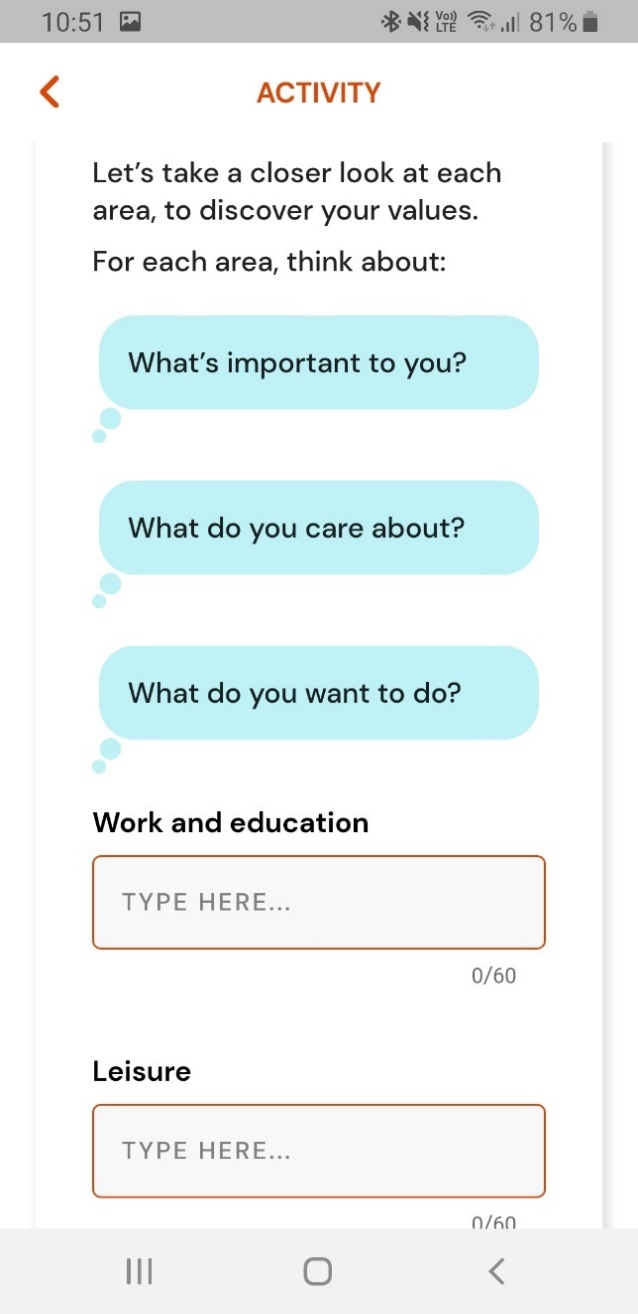

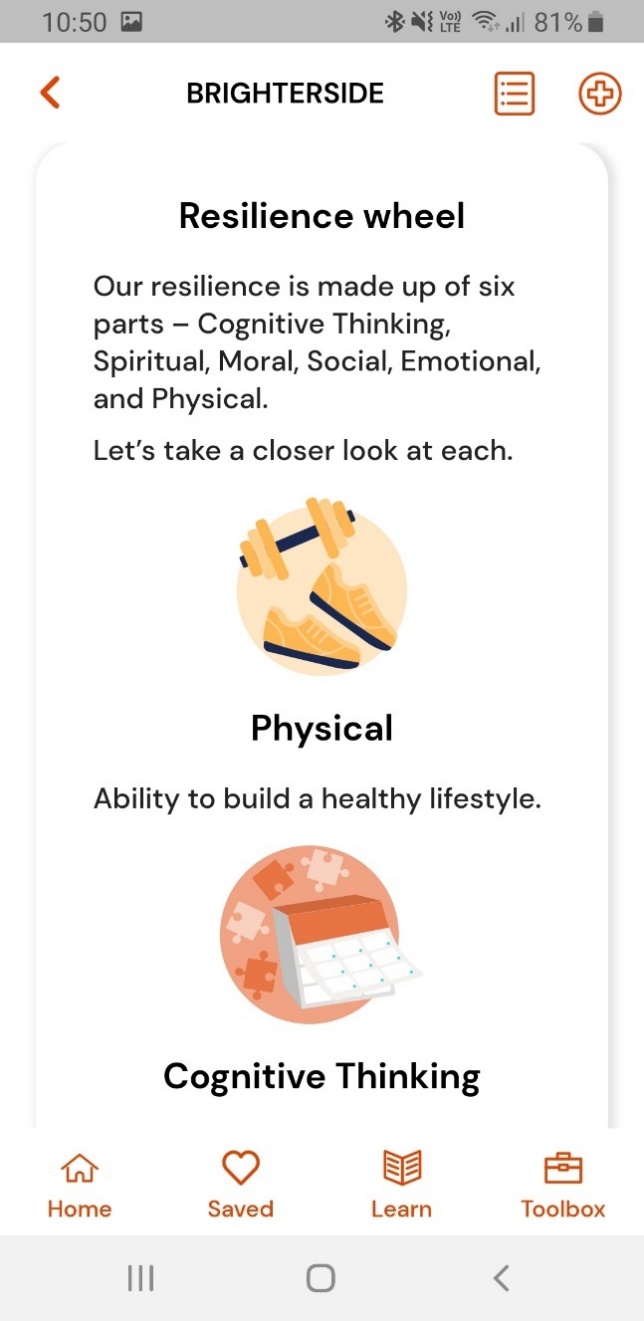

Supplement: Multimedia Appendix 2 [file mental-v11-e55528-s002.docx]
